# Expansive spatial pattern of Aβ deposition in patients with cerebral amyloid angiopathy: A three-dimensional surface-to-depth analysis

**DOI:** 10.1126/sciadv.aea7539

**Published:** 2026-02-06

**Authors:** Hideki Hayashi, Rie Saito, Akinori Miyashita, Takeshi Ikeuchi, Mari Tada, Kohei Akazawa, Osamu Onodera, Kazuki Tainaka, Akiyoshi Kakita

**Affiliations:** ^1^ Department of Pathology, Brain Research Institute, Niigata University, Niigata, Japan.; ^2^ Department of Neurology, Brain Research Institute, Niigata University, Niigata, Japan.; ^3^ Department of Molecular Genetics, Brain Research Institute, Niigata University, Niigata, Japan.; ^4^ Department of Medical Informatics, Niigata University Medical and Dental Hospital, Niigata, Japan.; ^5^ Department of System Pathology for Neurological Disorders, Brain Research Institute, Niigata University, Niigata, Japan.

## Abstract

Cerebral amyloid angiopathy (CAA) is a neurodegenerative condition characterized by amyloid-β (Aβ) deposition in small vessel walls, often coexisting with Alzheimer’s disease due to impaired Aβ clearance. However, the spatial distribution of Aβ within the human brain remains unclear as the vascular network’s complexity and scale hinder visualization by conventional thin-slice analysis. To address this, we performed three-dimensional (3D) volumetric imaging of the cerebrovascular network and Aβ deposition in autopsied brains with CAA using advanced tissue clearing and light-sheet fluorescence microscopy, labeling for smooth muscle actin (SMA) and Aβ. We found prominent Aβ deposition and SMA loss in leptomeningeal and superficial cortical segments, which were anatomically contiguous with deeper Aβ-positive segments, indicating a surface-to-deep progression pattern of Aβ extension. The perivascular plaque density was significantly lower around Aβ-positive vessels. This technology may provide further insights into CAA pathology and is recommended for research on the 3D pathology of neurological disorders.

## Teaser

3D imaging of CAA autopsy brains showed that vascular Aβ deposition can progress from the brain surface to peripheral vessels.

## INTRODUCTION

Cerebral amyloid angiopathy (CAA) is a disorder characterized by accumulation of amyloid in the cerebrovascular walls and brain parenchyma, being classified into hereditary and common sporadic forms (*1*–*3*). In sporadic CAA, amyloid-β (Aβ) is deposited in the cerebrovasculature and is the primary component of senile plaques (SPs) in Alzheimer’s disease (AD). AD and CAA coexist in as many as 80 to 90% of affected patients (*2*), indicating a shared Aβ pathophysiology. Sporadic CAA is also a major cause of cerebral hemorrhage in the elderly and can be accompanied by a reversible inflammatory process known as CAA-related inflammation (CAA-RI) (*4*). In addition, CAA is linked to amyloid-related imaging abnormalities (ARIA), explaining symptoms such as vascular edema and microhemorrhages during anti-Aβ immunotherapy (*2*, *5*–*8*). Additionally, the condition is associated with the apolipoprotein E (APOE) genotype, and APOE ε4 is considered a risk factor for CAA and ARIA, particularly CAA-RI (*4*, *9*, *10*).

Autopsy and ultrastructural studies of patients with CAA have demonstrated that Aβ is deposited predominantly in the outer basement membrane of vascular smooth muscle cells (SMCs), where amyloid fibrils progressively substitute SMCs in the vascular wall, and vessels with abundant Aβ deposition often lack actin-positive SMCs (*11*, *12*). In the moderate or advanced stages of CAA, the media can be almost completely replaced by amyloid and fibrotic material, accompanied by smooth muscle loss and structural disorganization (*1*). These characteristic changes form the basis of classical histopathological grading systems for CAA and are closely associated with vasculopathic alterations, such as fibrinoid necrosis and an increased risk of hemorrhage (*13*–*15*). Histopathologically, CAA is classified into two types: CAA type 1, in which Aβ is primarily deposited in capillaries, and CAA type 2, in which cortical capillaries are spared of Aβ deposition (*10*). In CAA type 1 and advanced CAA cases, dyshoric changes have been reported, characterized by the spread of Aβ into the brain parenchyma surrounding Aβ-positive vessels (*16*–*18*). This deposition occurs predominantly in the occipital lobe and then spreads (*19*), initially affecting the leptomeningeal arteries (LMAs) and cortical arteries, with capillaries and veins being less frequently involved (*20*). Similarly, tracer injection studies of rat brains have demonstrated that interstitial fluid drains alongside the LMAs and cortical arteries to the extracranial lymph nodes (*21*, *22*). These findings suggest that CAA results from impaired clearance of amyloid, rather than its excessive production (*23*–*26*), although in conditions with increased *APP* dosage, such as *APP* gene duplication or Down syndrome (trisomy 21), Aβ overproduction may also accelerate its development (*27*, *28*). The glymphatic system and the intramural periarterial drainage pathway (IPAD) are well-known as clearance pathways within the brain parenchyma, and other clearance systems involving transport across the blood-brain barrier (BBB), phagocytosis, and enzymatic degradation are also thought to be involved (*25*, *29*). In this context, mouse model studies have demonstrated that the IPAD flows retrogradely out of the brain parenchyma along the outer membrane of the SMC, indicating a major role in CAA (*24*, *30*–*33*). In the context of the human brain, however, this concept remains debatable due to challenges in observing Aβ dynamics, especially in the cerebrovasculature.

Amyloid positron emission tomography imaging quantifies amyloid deposition in the whole brain of a living patient with AD but cannot provide information at the cellular level or visualize blood vessels (*34*, *35*). Conventional histopathology using thin paraffin sections has provided important insights into the mechanisms of CAA, including leptomeningeal predominance and superficial-to-deep fading of Aβ deposition (*18*, *20*). However, such two-dimensional (2D) analyses are inherently limited to cross-sectional views of fragmented vascular branches, making it difficult to reconstruct the entire course of individual vascular units or to quantify a contiguous pattern of Aβ distribution along the vasculature. Thus, although the concept that CAA begins superficially is supported by previous studies, comprehensive 3D and quantitative visualization of its progression along the whole vasculature, from the leptomeninges to the white matter, has remained insufficient.

To address this issue, we have developed an innovative 3D imaging technique enabling single-cell resolution and stereoscopic assessment of large brain tissue volumes, including the lipid-rich white matter and successfully visualized the complex vascular network using light-sheet fluorescence microscopy (LSFM) (*36*, *37*). A few studies have applied this methodology to human brain samples, investigating neurological disorders or CAA in single patients (*8*, *38*, *39*), with only limited attempts to examine amyloid-vasculature correlations across a series of patients. In the present study using postmortem brains, we used this technique to clarify the spatial deposition pattern of amyloid in the cerebrovasculature, the pathological characteristics of vascular units showing amyloid deposition, and the quantitative relationship between perivascular SPs and vascular deposition of amyloid.

## RESULTS

### 3D visualization and characterization of the cerebral vascular network in the CAA

**Fig. 1.**
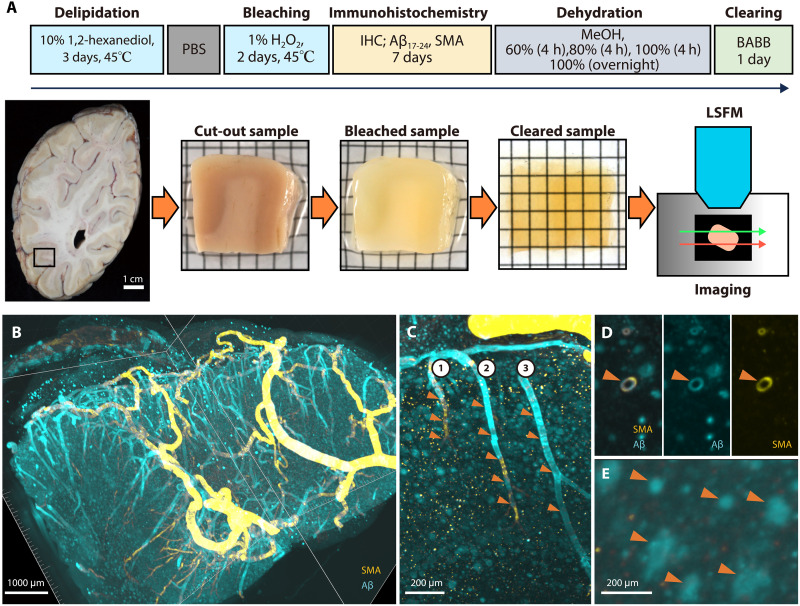
Tissue clearing protocol and 3D visualization of the cerebral vascular network in CAA. (**A**) Tissue clearing protocol: Brain samples were cut from 1-cm-thick occipital lobe slices corresponding to Brodmann areas 18 and 19 after 10% formalin fixation, followed by delipidation and bleaching. Each sample was double-immunostained with antibodies against smooth muscle actin (SMA) and Aβ_17-24_, dehydrated, and cleared with benzyl alcohol and benzyl benzoate (BABB). The samples were photographed using LSFM. (**B**) Patient 6, frontal lobe. 3D reconstruction of the vascular network. SMA (yellow) and Aβ (blue). The magnified images clearly demonstrate: (**C**) three vascular units (1 to 3) with Aβ deposition and loss of SMA (arrowheads), with numerous yellow dot-like structures, which are neuronal lipofuscin artifacts, (**D**) a cross section of a vascular unit with Aβ deposition and preserved SMA (arrowheads), and (**E**) SPs in the brain parenchyma (arrowheads). PBS, phosphate-buffered saline; IHC, immunohistochemistry; MeOH, methanol; h, hours.

We performed an advanced tissue clearing technique on brain samples (frontal lobe and occipital lobe) from six patients with CAA (Fig. 1A). For detailed information about this advanced tissue clearing technique and clinicopathological data of these patients, please refer to Materials and Methods and table S1.

3D imaging using immunostaining for smooth muscle actin (SMA) and Aβ detected arteries and arterioles with a diameter of ≥8 μm from the brain surface to the white matter with Aβ deposits in the vessel walls (Fig. 1B). We defined a “vascular unit” as an anatomically continuous vessel tree branching from a common LMA. In some vascular units, predominant Aβ deposits and segmental loss of SMA were clearly observed, and dyshoric changes were distributed along some of these Aβ-positive vessels. SPs were also visualized as spotty structures in the cortex. We confirmed the overall validity of the 3D imaging data by comparing them with adjacent thin sections processed by conventional Aβ immunohistochemistry. Although individual plaques were not matched one-to-one between the 2D and 3D datasets, thin-section immunohistochemistry showed that regions with predominantly diffuse plaques tended to exhibit faint Aβ signals, whereas regions with a higher proportion of neuritic plaques presented stronger immunoreactivity. A similar trend was observed in the 3D volumetric reconstructions, where variations in plaque composition appeared as differences in signal intensity. These intensity differences were often more accentuated in the rendered volumetric views compared to the cross-sectional 3D images (Fig. 1, B to E; movies S1 and S2; and fig. S1). A total of 1639 cerebral vascular units consisting of arteries and arterioles were included in the 12 samples used for analysis (865 vascular units in the frontal samples and 774 in the occipital samples). Of these, 1104 vascular units were assessed as Aβ positive. In the frontal samples, 513 of the 865 vascular units were Aβ positive (59.3%), and, in the occipital samples, 591 of the 774 vascular units (76.4%) were Aβ positive. The vascular Aβ load classification based on the percentage of Aβ-positive vascular units identified two samples as low, four as moderate, and six as high (table S1).

### Aβ deposition sites in the cerebral vascular network: Predominance in superficial segments

**Fig. 2.**
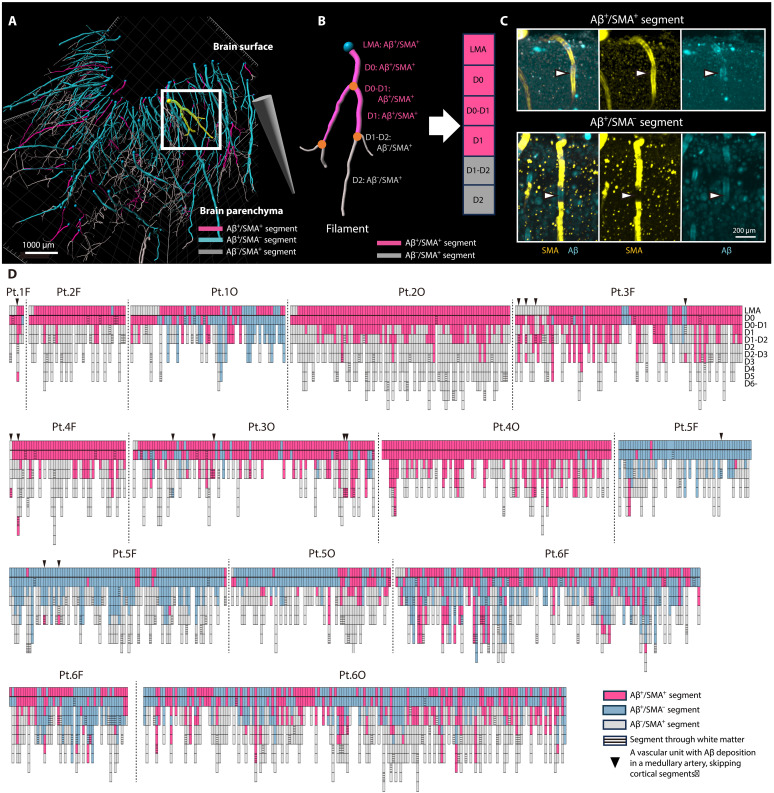
Patterns of Aβ deposition and SMA loss in each segment of Aβ-positive vascular units. Workflow for segmentation and classification. (**A**) Patient 6, frontal lobe. A schematic 3D vascular network shows Aβ^+^/SMA^+^ (pink), Aβ^+^/SMA^−^ (blue), and Aβ^−^/SMA^+^ (gray) segments. The point where arterioles enter the parenchyma is indicated by a blue dot. The cone tip denotes deeper parenchyma. (**B**) Magnified view of the yellow-highlighted vascular unit. Segments were categorized semiquantitatively as Aβ^+^/SMA^+^ or Aβ^−^/SMA^+^. The LMA segment was defined as the portion immediately above the parenchymal entry point, and its Aβ deposition and SMA loss were included in the analysis. (**C**) Determination of SMA loss in Aβ^+^ segments. (**D**) Segmental distribution of Aβ deposition and SMA loss in vascular units, arranged by vascular Aβ load. Boxes indicate Aβ^+^/SMA^+^ (pink), Aβ^+^/SMA^−^ (blue), and Aβ^−^/SMA^+^ (gray). The thick black line marks the brain surface; striped shading denotes segments within the corticomedullary junction or white matter. A triangle marks a vascular unit in which Aβ first appears in a medullary artery skipping cortical segments. Pt., patient; F, frontal lobe; O, occipital lobe; Aβ^+^, Aβ-positive; Aβ^−^, Aβ-negative.

To create a comprehensive map of the complex vascular network, arteries and arterioles within the brain parenchyma were traced from the obtained 3D images. Each vascular unit was then systematically segmented and labeled from depth 0 to depth 6 (D0 to D6) according to the order of branching from the cortical surface to the white matter. Furthermore, vessel segments with Aβ deposition and SMA loss were evaluated within each vascular unit (Fig. 2, A to C).

**Fig. 3.**
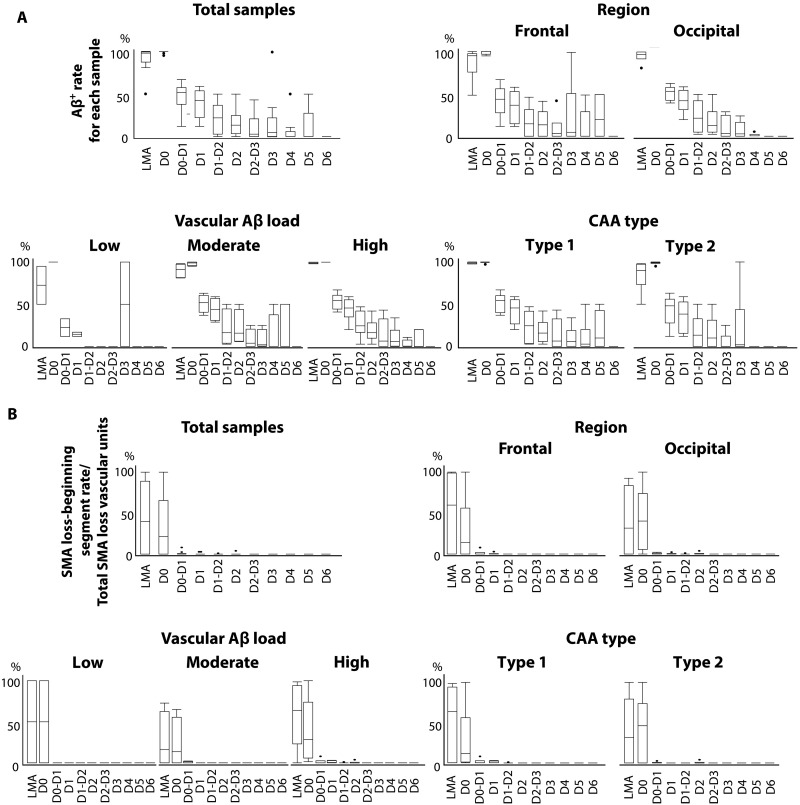
Quantitative analyses of vascular Aβ load and SMA loss. (**A**) Vascular Aβ load rate per segment by total samples, region, vascular Aβ load group, and CAA type. (**B**) Percentage of segments showing initial SMA loss by total samples, region, vascular Aβ load group, and CAA type.

In the 1104 Aβ-positive vascular units, Aβ deposition was observed at a high rate in LMAs just before bifurcation into the parenchyma and in the D0 segment (vascular Aβ load rate 96.2% in the LMA and 99.5% in the D0 segment). The vascular Aβ load of the D1 segment was markedly reduced to almost half that of the D0 segment, while the adjacent upper bifurcation, the D0-D1 segment, was highly positive, as was the case in the D0 segment (D0-D1 segment, 97.9%; and D1 segment, 45.8%). The vascular Aβ load rate then decreased toward the deeper segments (D2 segment, 19.8%; D3 segment, 10.0%; D4 segment, 4.5%; D5 segment, 6.3%; and deeper segments from D6, 0%) (Figs. 2D and 3A). Subanalysis of vascular Aβ load in each segment in terms of region, vascular Aβ load, and CAA type also yielded similar results, with Aβ deposition consistently concentrated in the vascular segments close to the brain surface (Fig. 3A). Vascular units commonly exhibited an anatomically continuous vascular Aβ load from the superficial to the deeper segments, although the presence of Aβ within each segment was sometimes sporadic. In contrast, 11 vascular units exhibited an unusual Aβ deposition pattern confined to deeper segments with skipping of cortical segments; such exceptional units also had Aβ deposition in the LMA or D0 segments. This unusual pattern was observed even in samples with mild deposition, regardless of vascular Aβ load.

### Distribution of vascular segments with SMA loss: Correlation with sites of Aβ deposition

Vascular units with SMA loss accounted for 547 of the total 1104 samples. Among the Aβ-positive vascular units, the median percentage of the most superficial segment exhibiting SMA loss was 40% for LMA, 21% for D0, and 0% for D1. In the deeper segments, vascular SMA was well preserved (for most superficial segments exhibiting SMA loss: median, 0% for segments deeper than D1), and, when SMA loss was present in the deeper segments, the superficial segments also showed SMA loss (Figs. 2D and 3B). These findings suggest that SMA loss is predominant in superficial segments of the brain, exhibiting a pattern similar to the anatomically continuous Aβ deposition that extends along connected vascular segments from the surface to deeper parts of the brain.

### Changes in the size of vascular units with Aβ deposition

**Fig. 4.**
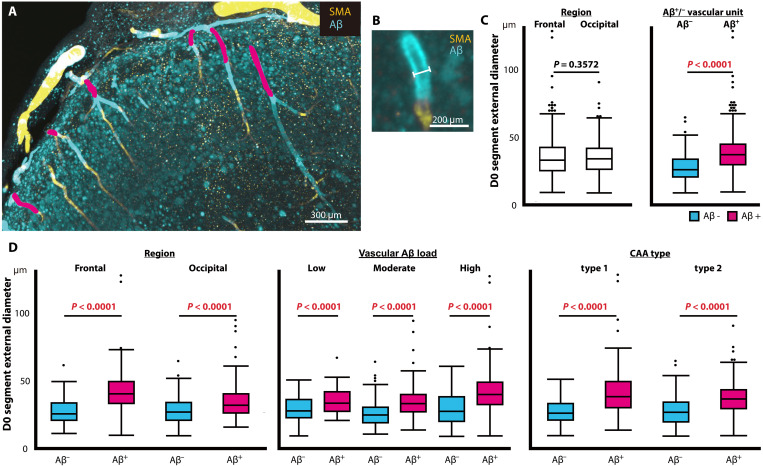
Distribution of the external diameter of the D0 segment in vascular units. (**A**) Definition of the external diameter. Filament widths (right, white line) are aligned with the D0 segments (left, pink areas). (**B**) External diameter of the D0 segment of vascular units in brain parenchyma. (**C**) External diameters in terms of region and Aβ-positive and Aβ-negative vascular units. (**D**) External diameter of Aβ-positive and Aβ-negative vascular units in terms of region, vascular Aβ load, and CAA type.

The external diameters of the D0 segments of each vascular unit were compared (Fig. 4, A and B). A complete list of the external diameters of the D0 segments in all cases is provided in data file S1. Frontal and occipital samples showed no significant difference in the maximum external diameter (D0 segment) of acquired vascular units, with a median (25th to 75th percentiles) of 32.72 μm (25.09 to 42.03 μm) and 33.85 μm (26.08 to 41.36 μm), respectively (*P* = 0.357) (Fig. 3C). On the other hand, Aβ-positive vascular units had significantly larger external diameters than Aβ-negative vascular units, with a median of 36.73 μm (29.41 to 44.45 μm) and 25.94 μm (20.47 to 33.39 μm), respectively (*P* = 1.73 × 10^−67^) (Fig. 4C). Subanalysis in terms of region, vascular Aβ load, and CAA type showed that Aβ-positive vascular units had a significantly larger external diameter than Aβ-negative units (frontal lobe, *P* = 2.32 × 10^−48^; occipital lobe, *P* = 7.20 × 10^−21^; vascular Aβ load-low, *P* = 7.44 × 10^−5^; moderate, *P* = 4.06 × 10^−28^; high, *P* = 5.89 × 10^−14^; CAA type 1, *P* = 2.12 × 10^−24^; and CAA type 2, *P* = 8.52 × 10^−14^) (Fig. 4D).

### Perivascular plaque density

**Fig. 5.**
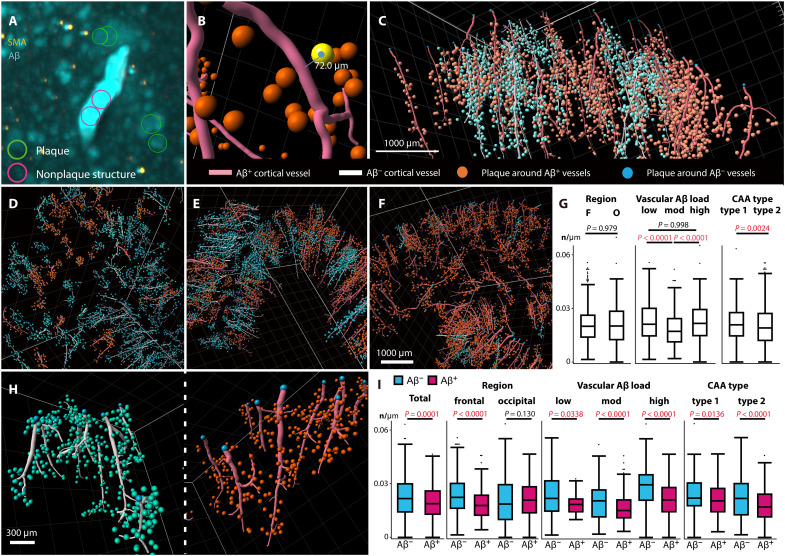
Large-scale perivascular plaque density around Aβ-positive and Aβ-negative vascular units. (**A** to **C**) Analytical workflow. (A) Machine learning to identify perivascular SPs in the range of 10 to 100 μm from a vessel (green circles). Nonplaque structures are circled in pink, such as Aβ deposits on vessels (top). Volumetric double immunostaining for SMA (yellow) and Aβ (blue). (B) Perivascular SPs converted to spots color-coded (orange spheres) and vascular filaments (red lines). Distance between the center of a plaque (colored yellow) and the vascular filament is indicated as an example. (C) Traced filaments representing Aβ-positive (red lines) and Aβ-negative vascular units (white lines) with perivascular spots in a sample. Representative large-scale images of vascular units and perivascular plaques in a single case from (**D**) low (Pt.2 frontal lobe), (**E**) moderate (Pt.3 frontal lobe), and (**F**) high (Pt.6 frontal lobe) groups. (**G**) Perivascular plaque densities in terms of region, vascular Aβ load, and CAA type. (**H**) Magnified images of perivascular plaques around Aβ-positive (right) and Aβ-negative (left) vascular units from a high case (Pt.5 occipital lobe). (**I**) Subanalysis of perivascular plaque densities around Aβ-positive and Aβ-negative vascular units, shown in terms of total sample, region, vascular Aβ load, and CAA type.

The numbers of plaques around individual vascular units, labeled with anti-Aβ antibodies in the brain parenchyma, were compared (Fig. 5, A to C). For assessment of perivascular plaque, among a total of 1639 vascular units, we excluded 299 that were unsuitable for analysis because of artifacts in the perivascular brain parenchyma. The artifacts excluded from the perivascular plaque analysis were those in which the blue-labeled puncta identified as plaques were also labeled with SMA at comparable intensity; these were regarded as autofluorescence artifacts and therefore excluded from analysis. The total length of vascular units was 2.36 × 10^5^ μm for individual units with a median (25th to 75th percentiles) length of 1.55 × 10^3^ μm (1.02 to 2.31 × 10^3^ μm), and the total number of perivascular plaques obtained was 4.72 × 10^4^ for individual units with a median of 30 (19 to 45). By region, the perivascular plaque densities for each sample in the frontal and occipital lobes were 0.0200 number (n)/μm (0.0139 to 0.0261 n/μm) and 0.0202 n/μm (0.0126 to 0.0284 n/μm), respectively, with no significant difference (*P* = 0.9707). Perivascular plaque density was compared across vascular Aβ load groups using the Kruskal-Wallis test (*P* = 6.17 × 10^−12^) followed by the Steel-Dwass test. Although the number of perivascular plaques was higher for samples showing greater vascular Aβ load, the low and high groups had significantly greater perivascular plaque density than the moderate group [low, 0.0211 n/μm (0.0146 to 0.0300 n/μm); moderate, 0.0171 n/μm (0.0112 to 0.0243 n/μm); high, 0.0216 n/μm (0.0147 to 0.0294 n/μm); low versus moderate, *P* = 4.72 × 10^−6^; and high versus moderate, *P* = 2.05 × 10^−11^]. There was no significant difference in perivascular plaque density between the low and high groups (low versus high, *P* = 0.998). In terms of CAA type, perivascular plaque densities were significantly greater for CAA type 1 than for CAA type 2, with a median of 0.0208 n/μm (0.0146 to 0.0276 n/μm) and 0.0191 n/μm (0.0121 to 0.0272 n/μm), respectively (*P* = 0.0024) (Fig. 5, D to G).

The median density of perivascular plaques was significantly higher around Aβ-negative vascular units [0.0218 n/μm (0.0142 to 0.0300)] than around Aβ-positive units [0.0189 n/μm (0.0131 to 0.0261); *P* = 0.0001]. Unexpectedly, the perivascular plaque density around Aβ-negative vascular units was significantly higher than that around Aβ-positive units. Subanalysis in terms of region, vascular Aβ load, and CAA type revealed that the perivascular plaque density was significantly higher around Aβ-negative vascular units; however, this observation was found only in the frontal lobe, and not in the occipital, in all groups (Fig. 5, H and I).

Dyshoric change, which often occurs around the vessel wall and exhibits a predominantly linear deposition pattern (*19*, *20*), was not detected by our plaque-spot detection algorithm because it does not have a spherical profile and was therefore not included in the analysis of plaque density. Accordingly, we evaluated the frequency of dyshoric change within Aβ-positive vascular units and determined which vascular segments (depths) were affected.

Dyshoric change was observed in six samples: all from the frontal and occipital lobes of patients 1, 4, and 5 (fig. S2). Among the Aβ-positive vascular units analyzed for perivascular plaque density, those extending into the white matter were excluded in advance. Accordingly, dyshoric change was evaluated in 465 cortical vascular units that were confined to within the cortex. For each sample, we counted the number of vascular units with accompanying dyshoric change and calculated the proportion of these units relative to the total number of cortical Aβ-positive vascular units within that sample. Overall, dyshoric change-positive vascular units accounted for 12.7% of the 465 cortical vascular units analyzed. The proportion of dyshoric vascular units ranged from ~3 to 67% across samples [patient 1 frontal lobe (Pt.1F), 66.7%; patient 1 occipital lobe (Pt.1O), 23.6%; Pt.4F, 27.0%; Pt.4O, 24.3%; Pt.5F, 3.4%; and Pt.5O, 13.6%]. Validation using adjacent conventional 2D Aβ-immunostained sections confirmed that the frequencies were comparable across samples. Regarding the depth distribution, dyshoric changes were most frequently observed in D0 segments, comprising 91.5% of all affected segments.

## DISCUSSION

In this study, we applied 3D volumetric imaging to reconstruct the entire vascular architecture and associated Aβ deposition in human brains with CAA. This approach enabled direct visualization of Aβ distribution along anatomically continuous vascular units, which cannot be achieved by conventional 2D histology.

Consistent with earlier pathological observations, Aβ deposition was most prominent in leptomeningeal and superficial cortical segments and diminished gradually toward deeper parenchymal branches (*18*, *20*). Notably, vascular units in which Aβ deposition appeared only in distal parenchymal segments without superficial involvement were extremely uncommon. These findings reinforce the concept of superficial-to-deep progression of CAA (*14*) while providing spatially contiguous and quantitative evidence at the whole-vascular-unit level.

Similarly, our 3D analysis also facilitated spatially continuous and quantitative assessment of SMA loss along individual vascular units. Although SMA loss in Aβ-laden vessels is a well-known feature of CAA, our results extend 2D histopathological observations (*11*–*15*) by clarifying its spatial distribution and anatomical continuity across the entire vasculature and the fact that external vessel diameters in Aβ-positive segments are larger than those in Aβ-negative segments.

The apparently larger diameter of Aβ-affected vessels observed here may be attributable to both pathological and methodological factors described in previous studies. Although one report has suggested that CAA vessels may appear to be thinner than non-CAA vessels (*12*), most studies have shown that the vascular media become thickened and distorted in the advanced stages of CAA (grades 2 to 4), often accompanied by double-barrel formation or fibrinoid necrosis, which can make the vessel diameter appear larger (*13*). In addition, because Aβ deposition begins at the abluminal side of the tunica media and gradually expands to fill it, and SMA labeling delineates the media, our measurements likely reflect the outer boundary of the media, thus making the diameter of Aβ-affected vessels appear larger. In this context, it is worth pointing out that perivascular dyshoric Aβ deposits adjacent to CAA vessels may not always be clearly distinguishable from the vessel wall in 3D images and, in some cases, may be included in the measurement of vessel diameter or vascular Aβ load, thus representing a methodological limitation. As vessels were classified dichotomously (Aβ positive or negative) irrespective of the precise distribution or amount of deposition, this potential overlap could have introduced a systematic bias, which we acknowledge as a study limitation.

Analysis of parenchymal Aβ deposits further revealed that SPs were observed less frequently around Aβ-positive than around Aβ-negative vascular units. By contrast, along Aβ-positive vascular segments, dyshoric perivascular Aβ deposition was occasionally observed, appearing as elongated stretches of Aβ accumulation in the parenchyma rather than as globular SPs.

The brain surface predominance of Aβ deposition in CAA, along with the anatomical continuity of vascular segments showing Aβ deposition from superficial to deeper segments, suggests spread from superficial to deep layers of the cerebral cortex. Several important observations support these ideas. In Aβ-positive vascular units, the deposition rate was highest in the LMA and the most superficial cortical segment (D0), showing 96.2 and 99.5% positivity, respectively. This rate decreased sharply toward deeper segments (45.8% in the D1 segment, with further reduction in the deeper segments). Moreover, vascular units showing Aβ deposition exclusively in the deep layers were extremely rare (less than 0.7% of all units), and such unusual deposition patterns were always characterized by superficial deposition. These findings argue against progression from deep to superficial layers or a random distribution. The consistency of this pattern across different brain regions, degrees of CAA severity, and CAA types may further suggest that a common mechanism of Aβ deposition underlies CAA. Similar findings have been obtained in mouse models of CAA, where Aβ deposition was observed in arteries on the brain surface from the early stages of the disease and then expanded to peripheral branches over time (*40*, *41*). In the brains of affected patients, while Aβ deposition in LMAs can be identified early through conventional 2D analyses of postmortem samples (*2*, *18*, *20*, *42*), it is unclear whether this deposition develops sporadically or extends in an anatomically continuous manner along a specific direction. Building on these findings, our study provides the detailed characterization of this process in the human brains with unprecedented resolution, offering previously unidentified evidence that help bridge the gap between human pathology and mouse models. It also raises the question of why Aβ distribution follows this pattern.

The pathomechanisms underlying vulnerability to Aβ deposition in leptomeningeal and superficial cortical branches rather than in mid-cortical and medullary branches remain unknown. On the other hand, several distinct factors on the brain surface or in superficial cortical layers that differ from those within the deeper cortex may have an impact. First, the soluble fractions of total Aβs and Aβ40 primarily associated with vascular deposition are preferentially located in the leptomeninges rather than the brain parenchyma (*43*, *44*). In addition, the leptomeninges do not contain cellular components involved in Aβ clearance, such as neurons, microglia, astrocytes, and their terminal feet (*26*). Further studies are needed to understand whether these factors contribute independently or interactively to brain surface vulnerability in early CAA.

The spatially continuous deposition of Aβ from the brain surface to deeper vascular segments supports the presence of an anatomically continuous structural pathway, such as IPAD. Through the IPAD, interstitial fluid containing Aβ and other waste molecules is assumed to drain out of the brain parenchyma from the capillaries along the basement membrane of vascular SMCs (*3*, *30*). Thus, the brain surface where Aβ aggregates early in the vessels corresponds to the downstream part of the IPAD pathway. SMA loss in vascular nets was observed at the specific vascular segment where Aβ deposition occurs. Within Aβ-positive vascular units, the segment of SMA loss was also predominantly on the same surface side of the brain, where the external vessel diameter was significantly larger than that of Aβ-negative vascular units. It has been reported that damage to amyloid-substituted tunica media results in loss of vasoconstriction followed by dilation (*45*), which is consistent with the present observations. Based on these findings, the following mechanisms of Aβ progression in the vascular wall can be considered: (i) Aβ deposition in the superficial vascular branches corresponding to the downstream part of the IPAD gradually obstructs the drainage flow, including upstream parts of the vascular unit. (ii) Increased Aβ deposition causes a reduction of vascular SMCs in the same area, resulting in loss of vasoconstriction followed by dilation. In turn, this could result in (iii) further reduction of drainage capacity and (iv) acceleration of Aβ deposition in the vascular unit (*25*, *46*). Our observations suggested that this cascade rarely involved capillaries in deep layers located upstream of the IPAD. This could also be related to the fact that capillaries constituting that part of the IPAD are absent in the leptomeningeal or superficial layers, where the vascular density is much lower than in deep layers (*47*), suggesting that IPAD capacity may be more robust in the deep layers. Nonetheless, possible alternative mechanisms should also be acknowledged, including (i) the unique microenvironment of the brain surface, where structures such as the pia mater and subarachnoid space may potentially promote Aβ deposition; (ii) potential differences in BBB function between superficial and deep layers; and (iii) the possible involvement of the glymphatic system in Aβ clearance (*29*, *44*). Although these alternative explanations cannot be completely ruled out, the superficial-to-deep pattern and the IPAD hypothesis best explain our current observations. While recognizing the multiple systems involved in Aβ clearance, further research using a multifaceted approach is essential to clarify their influence and deepen understanding.

As with small arteries in the deep cortex, both previous reports and the present study confirmed that the amount of Aβ deposition in medullary arteries running through the white matter was low (*1*, *2*, *48*, *49*), and, if present, deposits were numerous in cortical segments. In contrast, a small number of vascular units had Aβ deposits confined to white matter segments (6.2%). This deposition pattern goes against the known flow of IPAD. Such exceptions were observed even in early cases, suggesting that this phenomenon may occur at any disease stage. The drainage pathway through the white matter branches is still largely unclear, and further data are needed to understand the mechanism of Aβ deposition in medullary branches.

In this study, to investigate the relationship of Aβ deposited in vessels to SPs in the parenchyma, we used an Aβ_17-24_ antibody, which can detect both vascular and parenchymal Aβ equally, rather than Aβ_1-40_, which is more specific to vascular Aβ. Although findings have varied across studies regarding the relationship between CAA severity and SP density—some reporting a positive correlation (*50*), while others a negative (*2*, *15*, *19*, *51*, *52*) or no correlation (*53*)—our study has provided one clear perspective. By focusing strictly on perivascular plaques, we have demonstrated a consistent inverse correlation: Perivascular plaque density was lower around Aβ-positive vascular units, regardless of the degree of CAA or anatomical location. Notably, however, some Aβ-positive vascular units exhibited prominent dyshoric changes, characterized by linear perivascular Aβ accumulation rather than discrete globular SPs. These dyshoric changes, considered an advanced form of CAA (*16*–*18*), were predominantly evident along superficial cortical vessels, consistent with the topography of vascular Aβ deposition revealed by our 3D analysis. Aβ is the main component of SP and CAA, but the length of Aβ peptides varies by lesion, with prominent aggregation of Aβ_1-42_ peptides in SP and Aβ_1-40_ peptides in CAA and dyshoric change (*16*, *25*, *26*, *43*). It has also been suggested that CAA may interact with neuritic plaques and increase tau accumulation (*54*). Although the cellular compartments from which these different forms of Aβ originate are unknown, their soluble fractions are in dynamic equilibrium and are constantly being produced, degraded, and drained from the brain, as mentioned above (*25*, *55*). Together, these findings support the idea that Aβ in the brain parenchyma moves into blood vessels and aggregates there, followed by the development of CAA pathology and dyshoric change, thereby reducing the number of SP in the perivascular area. These findings may also suggest impairment of the local Aβ clearance system including IPAD.

One limitation of this study was that the postmortem brains of patients with CAA were studied retrospectively, which restricted the region available for investigation, and the entire vascular network of the brain was not included. Capillaries and veins were not assessed quantitatively because of their weak SMA immunoreactivity, and their staining intensity was not sufficient to allow their walls to be delineated reliably under the imaging conditions that we used. Therefore, it was only possible to analyze arteries consistently. In addition, hemorrhagic lesions were excluded because of extensive tissue damage, preventing any assessment of the association between hemorrhage-related vascular changes and Aβ load. We were unable to obtain any information on SMA loss in vascular units without Aβ deposition because we examined the relationship between Aβ deposition and SMA loss within Aβ-positive vessels. Thus, it was unclear whether non-Aβ–related vascular pathology (e.g., atherosclerosis) may have contributed to SMA loss. A further limitation of this study was the quantification of perivascular plaque density. Because it was not technically feasible to accurately measure the 3D volume of the brain parenchyma surrounding each vascular unit, we normalized the number of detected SPs to the corresponding filament length (spots per micrometer). However, this length-based normalization would not have fully accounted for variations in perivascular tissue volume across vascular units and therefore may not have completely reflected the actual Aβ load in the surrounding parenchyma. In addition, this analysis did not take into account the density or intensity of individual SPs. Very small or diffuse Aβ deposits were excluded by the size threshold applied in the spot detection algorithm, and dyshoric perivascular Aβ deposition was not incorporated into the quantification. Therefore, this method does not directly measure the total parenchymal Aβ load but rather focuses on the spatial relationship between clearly defined SPs and the vascular structures, providing a complementary perspective on parenchymal Aβ pathology. This approach is also not suitable for assessing the vasculopathic severity of CAA but rather for evaluating its extension (i.e., the number of vessels involved).

In conclusion, we quantitatively analyzed the spatial organization of the cerebrovascular network in postmortem brains with CAA and demonstrated a major pattern of Aβ extension from superficial to deeper locations, following the anatomical continuity of vascular segments. Our findings reinforce previously proposed concepts and provide 3D evidence of how Aβ spreads along the vasculature. In addition, a lower density of globular plaques was observed around Aβ-positive vascular units. Future studies taking into account perivascular dyshoric change and diffuse parenchymal Aβ deposition will be required for a more comprehensive evaluation of total perivascular Aβ load. The present study has provided data useful for clarification of CAA pathogenesis in the human brain. It is anticipated that our cutting-edge approach will yield further insights into the spatial pathology of neurological disorders.

## MATERIALS AND METHODS

### Patients and human brain samples

The present study was approved by the Ethics Committee of Niigata University (G2015-1192). Written informed consent for autopsy including the use of tissues for research purposes was obtained from the patients’ families. Because clearer 3D images can be obtained from formalin-fixed samples with a fixation period of less than 5 years, we selected six individuals with a pathological diagnosis of CAA Thal stage 2 or 3 from autopsied patients examined consecutively at the Department of Pathology, Brain Research Institute, Niigata University, between 2017 and 2020 [four males and two females aged 79.5 ± 9.5 years (mean ± SD), range of 67 to 95 years] (*12*). The clinical and pathological profiles of the patients are summarized in table S1. Brain MRI data were available for five of the six patients, and T2-weighted imaging had demonstrated cerebral white matter hyperintensities in all of them; four of the five patients had enlarged perivascular spaces, and T2* images available for these four showed that three had multiple cerebral microbleeds (fig. S3). One of the six patients (patient 5) had cerebral lobar hemorrhage; in this case, tissue samples were prepared from regions without macroscopic hemorrhage (fig. S4).

### Histopathological analysis

The brains were fixed with 10% buffered formalin, and multiple tissue blocks were embedded in paraffin. Histopathological examination was performed on 4-μm-thick sections stained with hematoxylin and eosin, and the standard Klüver-Barrera method with Luxol fast blue and Nissl staining was also used for 2D observation of structural integrity. In addition, selected sections were immunostained with antibodies against Aβ_11-28_ (IBL, Gunma, Japan; 1:50; antigen retrieval, formic acid), phosphorylated tau (Fujirebio, Ghent, Belgium; 1:200), and phosphorylated α-synuclein (Wako, Saitama, Japan; 1:1000; antigen retrieval, formic acid) to assess CAA pathology, senile pathologic changes based on the “ABC” score, and the fourth consensus report of the DLB Consortium (*56*–*58*). The CAA Thal stage (*19*) was stage 2 in four patients and stage 3 in two. Three patients each with CAA type 1 and CAA type 2 were included (*9*). To evaluate vascular pathology, arteriolosclerosis was assessed using a conventional 2D method on the basis of a previous report (*59*, *60*).

### Tissue block clearance and 3D imaging of human brains

From each patient, we prepared 10% formalin-fixed 0.5-cm^3^ tissue blocks of the occipital lobe, where CAA is observed most frequently, and the frontal lobe (including the middle frontal gyrus), where CAA is observed less frequently (*19*). For the occipital samples, regions within Brodmann areas 18 and 19 showing good retention of LMAs were selected (fig. S4). The time from death to autopsy was 250 ± 116 min (range, 150 to 480 min), and the formalin fixation period was 2 to 3 years. The slices were adjusted to meet both antigenicity and clearing requirements on the basis of our tissue clearing protocols (*36*, *37*). To detect vascular nets and amyloid deposits in blood vascular units, we used an Alexa Fluor 647–conjugated antibody against α-SMA (Novus Biologicals, 1A4/asm-1; 1:100) and an Aβ_17-24_ antibody (BioLegend, SIG-39220; 1:100) labeled with HiLyte FluorTM 555 Labeling Kit - NH_2_ (Dojindo Molecular Technologies Inc.). The advanced tissue clearing protocol consisted of four steps: (i) delipidation, (ii) bleaching with H_2_O_2_, (iii) immunostaining, and (iv) clearing. Specifically, (i) cut-out samples were immersed in 10% 1,2-hexanediol diluted in distilled water at 45°C and shaken for three days. (ii) After washing with phosphate-buffered saline, the samples were again immersed in 1% H_2_O_2_ at 45°C and shaken for 2 days. (iii) The samples were placed in 1 ml of immunostaining buffer (a mixture of phosphate-buffered saline, 0.5% Triton X-100, 0.25% casein, and 0.01% NaN_3_) containing fluorescence-labeled anti–Aβ_17-24_ and anti–α-SMA antibodies. The samples were then shaken for 7 days under light-shielded conditions. (iv) The samples were dehydrated by shaking at room temperature and placing them in a series of methanol dilutions as follows: 60% (4 hours), 80% (4 hours), 100% (overnight), and 100% (4 hours). They were then placed in a 1:2 mixture of benzyl alcohol and benzyl benzoate containing 3% *N*-butyl diethanolamine and shaken overnight at room temperature to clear the samples. 3D images of SMA- and Aβ-positive structures were captured using LSFM. We used two different LSFM systems, the Olympus MVX10-LS and the UltraMicroscope Blaze (Miltenyi Biotec), to accelerate image acquisition. To validate comparability, we imaged the same sample with both instruments (fig. S5), and, as the appearance was essentially identical, we did not unify the imaging to a single method. The imaging conditions used were as follows: occipital lobe samples of patients 1, 3, and 5, Olympus, MVX10-LS. SMA, excitation of 637 nm; Aβ_17-24_, excitation of 532 nm; 0.63× objective lens, 1× digital zoom. Other samples: Miltenyi Biotec, UltraMicroscope Blaze. SMA: excitation of 640 nm; Aβ_17-24_, excitation of 561 nm; 1× objective, 0.66× digital zoom.

To verify the specificity of Aβ detection, a brain sample without detectable Aβ deposition was processed in the same manner. No Aβ-positive structures were observed in this negative sample (fig. S6).

### Quantification of arteries and arterioles and identification of Aβ deposition sites in the vascular network

Tiff images were converted to IMS files using Imaris File Converter 9.3.1 or 10.0.0.0 and reconstructed as 3D vascular images visualized with anti-SMA antibody, using Imaris 10.0.0 (Bitplane) image analysis software. To analyze the complex vascular network with numerous branches, we defined a vascular unit as an anatomically continuous vasculature formed by branching from a common LMA. Then, branches were traced and segregated by unit from the point of penetration into the parenchyma to the cortical or medullary branches using the Filament tool of Imaris (movie S3). We excluded any vascular units for which the intraparenchymal penetration position could not be confirmed from the brain surface. In a single vascular unit, each segment was systematically labeled from depth 0 to depth 6 (D0 to D6) according to the order of branches from the brain surface to the white matter, with D0 referring to the vascular segment from penetration into the cortex to the first branch and D1 referring to the segment from the end of D0 to the next branch; the bifurcation between D0 and D1 was labeled D0-D1. The LMA segment was defined as the starting point of each filament, corresponding to the portion of the vessel immediately before it enters the parenchyma (Fig. 2, A and B). The presence or absence of Aβ deposits in a vascular segment was evaluated semiquantitatively and defined as an Aβ-positive segment if present and an Aβ-negative segment if absent. We then assessed the “vascular Aβ load” of CAA in each frontal and occipital sample based on the density (%) of Aβ-positive vascular units among the total vascular units in one sample: low, <40%; moderate, 40 to 60%; and high, >60%. For each sample, the vascular Aβ load rate was also calculated for each segment, and the areas where Aβ was more likely to be deposited were evaluated.

### Distribution of vascular segments with SMA loss

To investigate the relationship between SMA loss and Aβ deposition, we examined the distribution of segments at a site where SMA had been lost and replaced by Aβ in Aβ-positive vascular units (movie S4). For each Aβ-deposited segment, the SMA-only channel was examined at the corresponding location. If the vascular structure could still be traced in the SMA channel, then the segment was classified as Aβ-positive/SMA-positive. If the vessel could not be traced in the SMA channel, then it was classified as Aβ-positive/SMA-negative. Most segments with SMA loss were contiguous from the superficial segment to the deeper segment, and few vascular units had SMA loss confined to the deeper segment only. Therefore, to evaluate the distribution of SMA loss in each Aβ-positive vascular unit, we focused on the most superficial segment of the brain exhibiting SMA loss. We identified this segment among D0-D6 in each vascular units with SMA loss and determined its proportion relative to the total number of vascular units showing SMA loss in each sample. The distribution of most superficial segments exhibiting SMA loss was then compared in terms of region, vascular Aβ load, and CAA type.

### Changes in the size of vascular units showing Aβ deposition

To examine changes in the size of vascular units showing Aβ deposition, we measured the external diameter of the D0 segment, which had the largest diameter of all the segments in a vascular unit labeled with anti-SMA, and compared the dimension of the external diameter for each of the Aβ-positive and Aβ-negative vascular units in terms of region, vascular Aβ load, and CAA type.

### Large-scale quantification of perivascular plaque density

To identify and count the number of perivascular SPs labeled with anti–Aβ_17-24_ antibodies in 3D images, we used the “Spot tool” from Imaris by replacing the plaques with spheres, referred to as “spots.” The spot diameter was set to 50 μm, and the “quality” range was set to 30 to 50 or higher. Using the deep learning function of the Spot tool, we adjusted the spots to detect plaques in the brain parenchyma, excluding those recognizing Aβ deposition within vascular walls and nonplaque structures. The distance of the spot from each filament created by tracing the vascular units was set at 10 to 100 μm (movie S5). The perivascular region analyzed was defined as the area within 10 to 100 μm from the surface of each vascular unit classified as Aβ-positive or Aβ-negative. This distance range was applied uniformly to all vascular units to standardize the analyzed volume. Because the density of perivascular plaques varies significantly, with more plaques in the cortex and fewer in the white matter, we excluded arteries penetrating the medulla (white matter) from this analysis and analyzed only cortical arteries. The number of spots calculated was divided by the total length of each filament (micrometers) to obtain the plaque density (number of spots per micrometer). The perivascular plaque densities around Aβ-positive and Aβ-negative vascular units were compared in terms of region, vascular Aβ load, and CAA type.

In addition, in this study, dyshoric change was defined as a linear parenchymal Aβ-positive structure running along the course of a vessel that was not detected as a “spot” by the plaque-spot identification algorithm but was located in close proximity to an Aβ-positive vascular unit. For each sample, we identified the number of vascular units exhibiting such dyshoric changes and determined which vascular segments (D0, D1, etc.) within these units were most frequently involved. These evaluations were performed by two neuropathologists (H.H. and R.S.).

### Statistical analysis

Quartiles [median values and (25th to 75th percentiles)] were used to represent the nonnormal distribution of continuous variables. Mann-Whitney *U* test was used for comparisons between two groups and Steel-Dwass test was used for multiple comparisons between three groups. Steel-Dwass multiple comparisons test was performed following Kruskal-Wallis test. Data were analyzed using the JMP Pro version 17.0.0 software package (SAS Institute). The significance level was set at *P* < 0.05.

## Supplementary Materials

### The PDF file includes:

Figs. S1 to S6 Table S1 Legends for movies S1 to S7 Legend for data file S1 References
