# Supplementary material for: Expansive spatial pattern of Aβ deposition in patients with cerebral amyloid angiopathy: A three-dimensional surface-to-depth analysis
Source: Sci Adv. 2026 Feb 6;12(6):eaea7539. doi: 10.1126/sciadv.aea7539 (PMC12880530; doi:10.1126/sciadv.aea7539)
Supplement: Supplementary file 1 — Figs. S1 to S6 Table S1 Legends for movies S1 to S7 Legend for data file S1 References [file sciadv.aea7539_sm.pdf]

Science Advances
AAAS

Supplementary Materials for

Expansive spatial pattern of Aβ deposition in patients with cerebral amyloid angiopathy: A three-dimensional surface-to-depth analysis

Hideki Hayashi *et al.*

Corresponding author: Rie Saito, riesaito@bri.niigata-u.ac.jp

Sci. Adv. **12**, eaea7539 (2026)
DOI: 10.1126/sciadv.aea7539

The PDF file includes:

Figs. S1 to S6
Table S1
Legends for movies S1 to S7
Legend for data file S1
References

Other Supplementary Material for this manuscript includes the following:

Movies S1 to S7
Data file S1

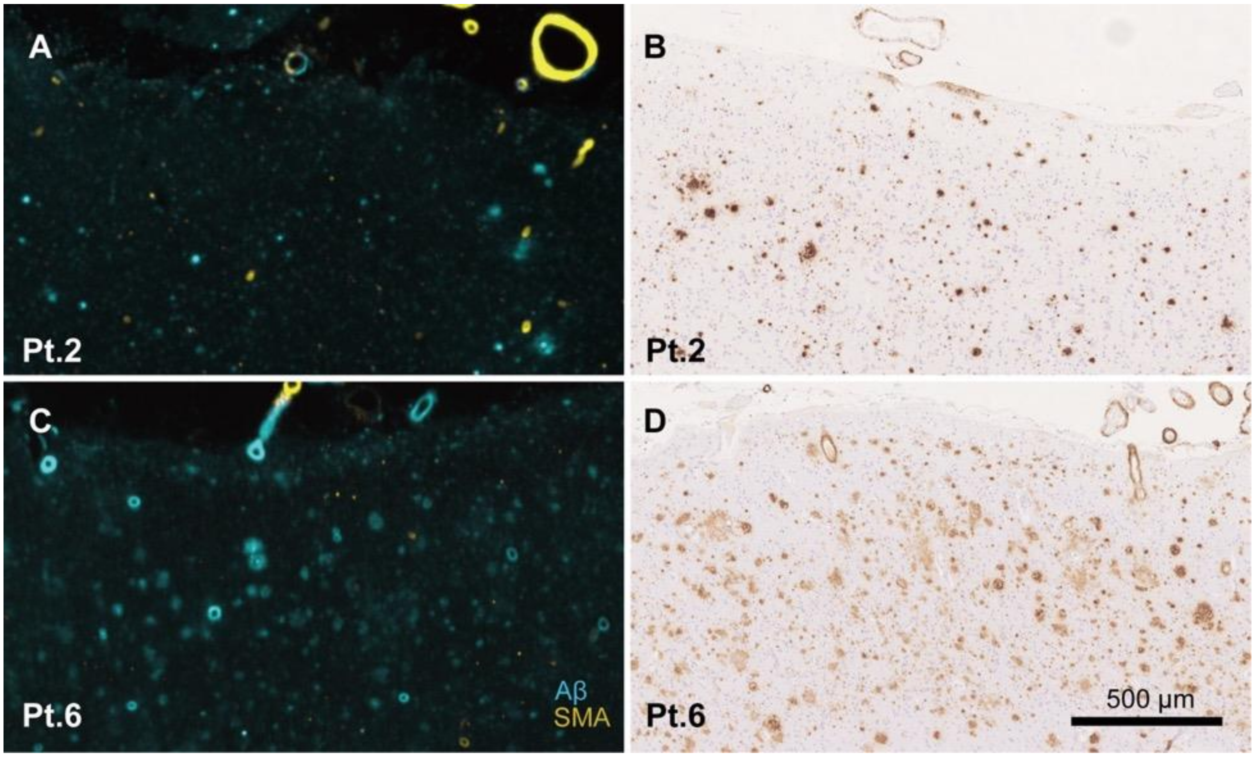

Fig. S1. Comparison of cross-sectional 3D images and adjacent conventional Aβ immunohistochemistry images. Representative images from Patient 2 and 6 (frontal cortex). When compared with the cross-sectional 3D merged images of Aβ and SMA in Patient 2 (**A**) and Patient 6 (**C**), Patient 2 showed predominantly neuritic plaques (**B**), whereas Patient 6 exhibited predominantly diffuse plaques (**D**) on the 2D immunohistochemistry image. (**C, D**) Aβ immunohistochemistry. Although the signal intensity of senile plaques varied depending on the plaque type, the number and spatial distribution of plaques were comparable between the cross-sectional 3D images and the adjacent 2D immunohistochemistry sections. Aβ, amyloid β; SMA, smooth muscle actin.

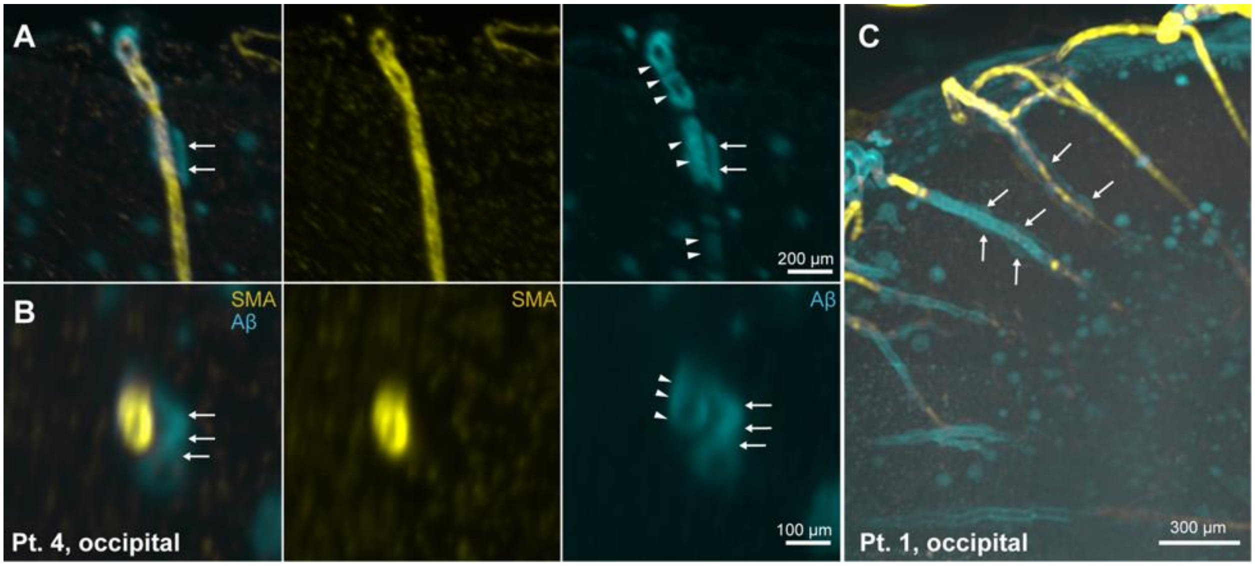

Fig. S2. Representative images of dyshoric change. Longitudinal (**A**) and cross-sectional (**B**) 3D imaging views of an occipital lobe sample from Patient 4. A: In the longitudinal view, linear Aβ deposition is evident in the parenchyma adjacent to Aβ-positive vascular segments (*arrowheads*), forming perivascular streaks of Aβ (*arrows*), referred to as dyshoric change. In the cross-sectional view (**B**), this dyshoric change appears as Aβ deposition surrounding the vessel wall within the adjacent parenchyma (*arrows*). Longitudinal 3D view of an occipital lobe sample from Patient 1 (**C**). Diffuse Aβ deposition is seen in the parenchyma around vessel segments located near the cortical surface (*arrows*), where few senile plaques are present in the surrounding parenchyma.

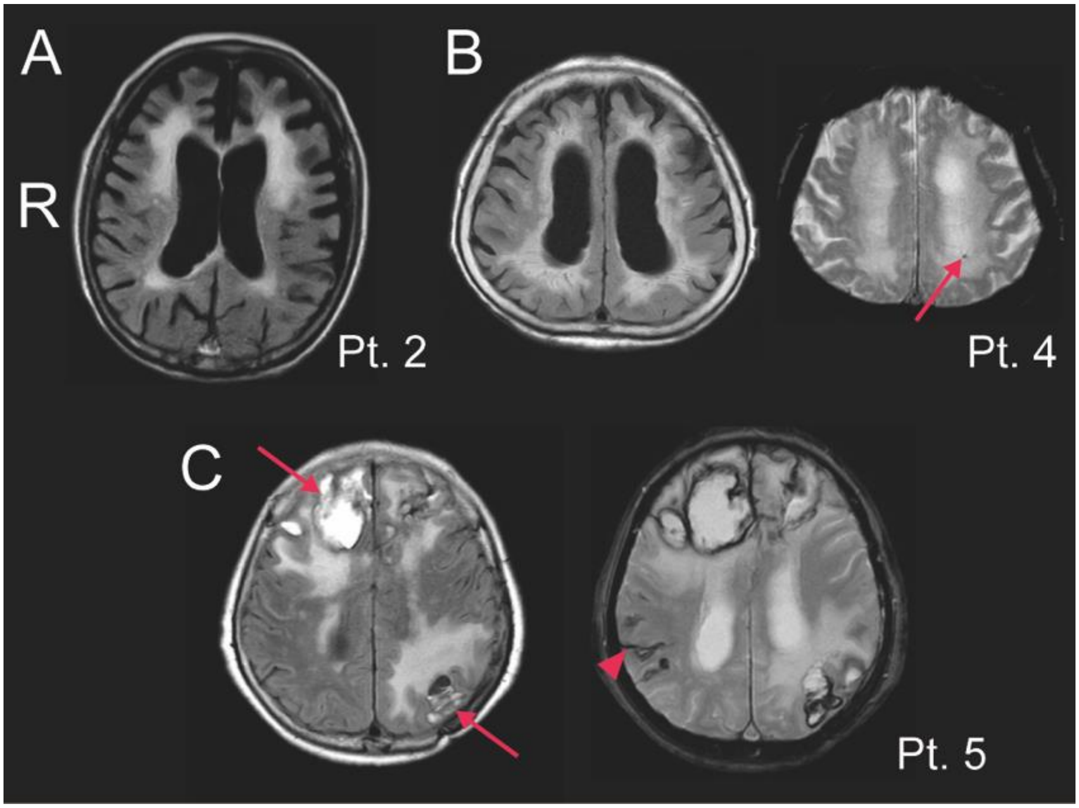

Fig. S3. Brain MRI images of atient 2, 4, and 5. MRI images of mild (A), moderate (B) and severe (C) cases of CAA. All cases exhibit cortical atrophy and high FLAIR signals in the deep white matter. In (B), microbleeds are evident (*arrow*), while in (C), lobar hemorrhagic lesions are present in the frontal and parietal lobes (*arrows*), as well as subarachnoid hemorrhage (*arrowhead*). (A, B, *left panel* and C, *right panel*) Fluid attenuated inversion recovery image. (B, *right panel* and C, *left panel*) T2*-weighted image. Pt, patient; R: right side of the brain.

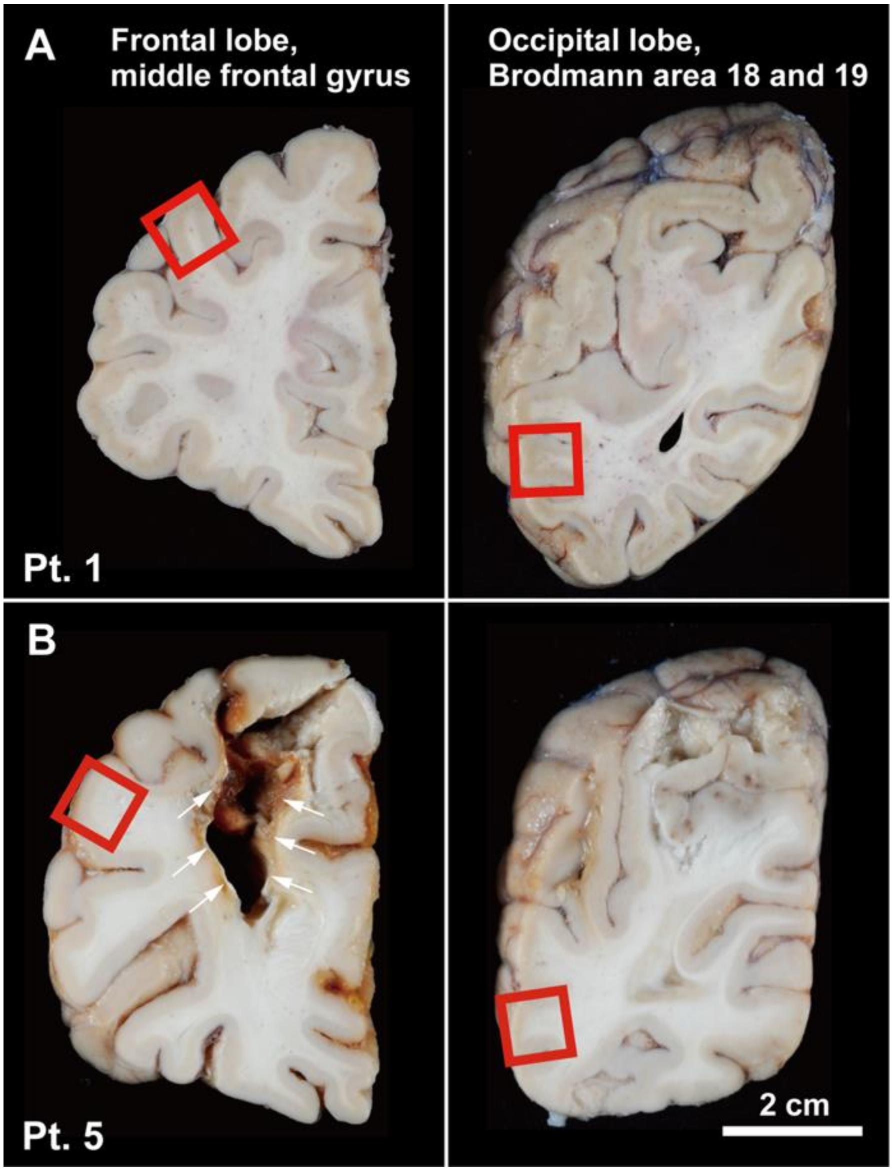

Fig. S4 Sampling site for 3D imaging in a formalin-fixed coronal brain slice. (**A**) Formalin-fixed coronal brain slices of the frontal and occipital lobes from Patient 1. The frontal samples from regions including the middle frontal gyrus, and the occipital samples, were taken from regions including Brodmann areas 18 and 19, and (*red squares*). (**B**) Brain slices from Patient 5 showing a cortical/subcortical intracerebral hemorrhage. A destructive lesion caused by hemorrhage is observed in the frontal lobe slice (*arrows*). The sample collection sites avoid areas with obvious hemorrhagic changes (*red squares*).

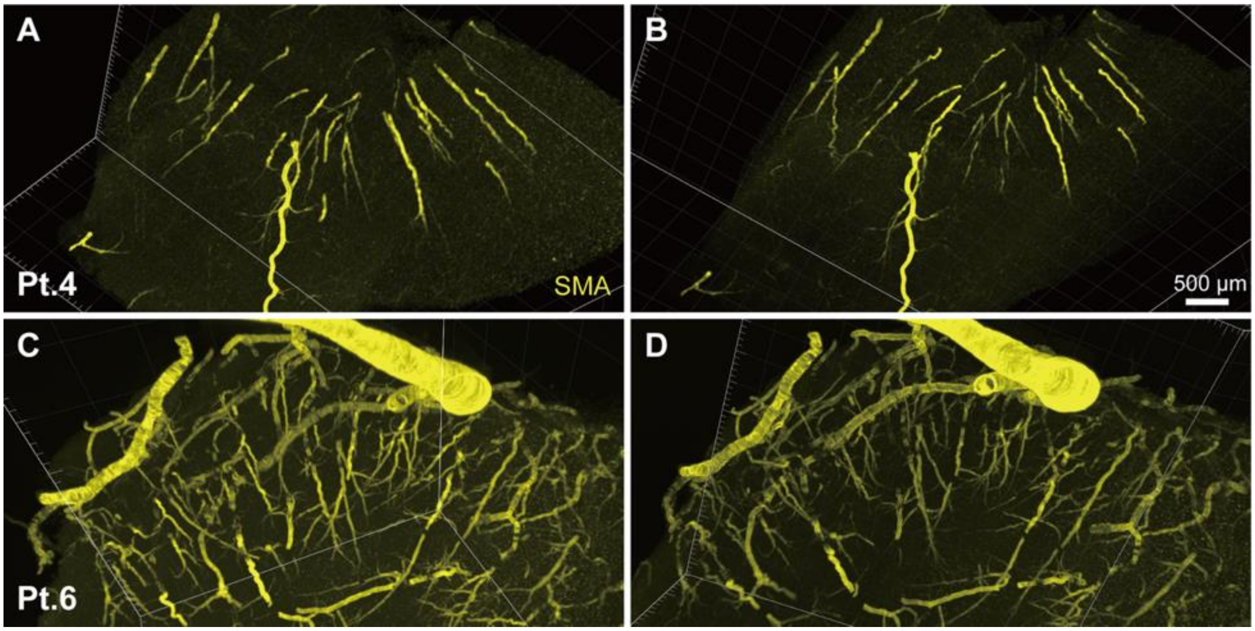

Fig. S5. Comparison of imaging data obtained using two light-sheet microscopy systems. Small frontal lobe samples from Patients 4 (**A, B**) and 6 (**C, D**) were imaged using both the UltraMicroscope Blaze™ (Miltenyi Biotec) (**A, C**) and the Olympus MVX10-LS light-sheet system (**B, D**) to validate comparability between the two setups. Vascular structures labeled with SMA (*yellow*) were visualized with no apparent differences in image quality or vessel delineation between the two systems, confirming that both protocols yield essentially identical results. Scale bar: 500 µm. SMA, smooth muscle actin.

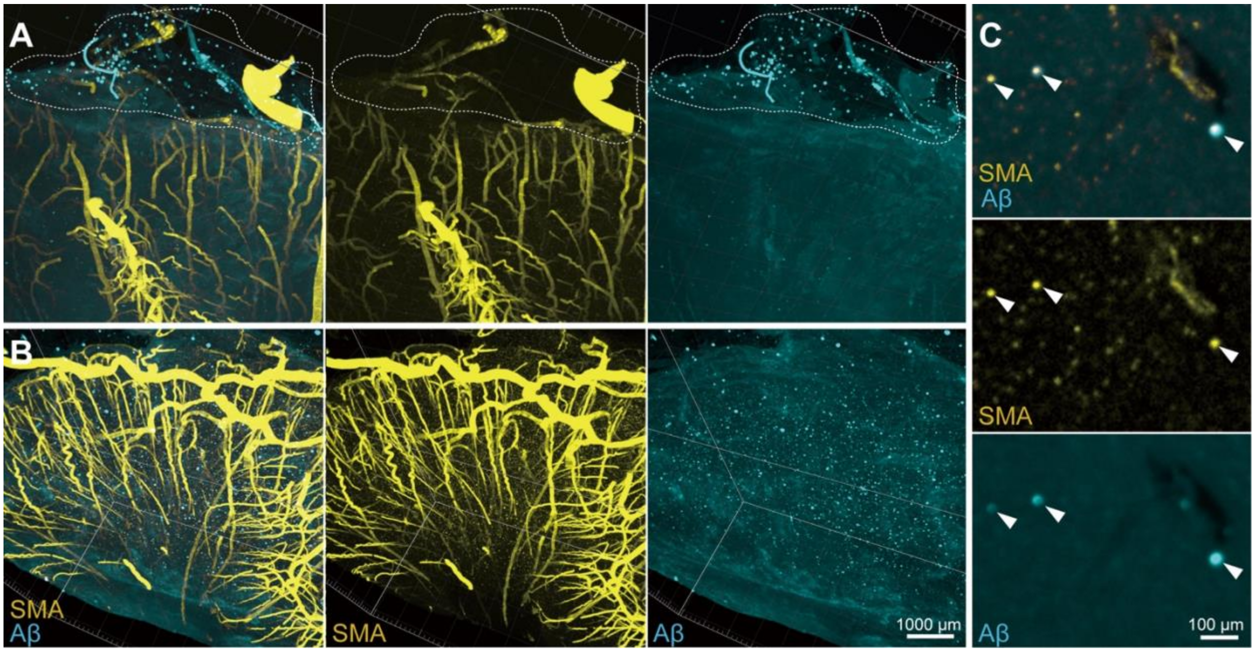

Fig. S6. 3D images of CAA-negative control patients. (A, B) 3D imaging data obtained from two CAA-negative control patients of Japanese origin. For each case, merged images of Aβ and SMA (*blue* and *yellow,* respectively), SMA-only images (*yellow*), and Aβ-only images (*blue*) are presented. **(A**) A 29-year-old male diagnosed with Duchenne muscular dystrophy. Braak (NFT) and Braak (SP) stage 0/0; AD neuropathologic change: A0B0C0 (Not). In the cross-sectional view derived from 3D imaging, punctate and linear Aβ-positive structures are evident along the brain surface (region indicated by a *dotted line*), representing non-specific staining, whereas almost no Aβ deposits are seen within the parenchyma. (**B, C**) An 83-year-old male diagnosed with Becker muscular dystrophy. Braak (NFT) and Braak (SP) stage II/0; AD neuropathologic change: A0B1C0 (Not). In the cross-sectional view derived from 3D imaging, numerous punctate high-signal structures are visible (**B**). However, these signals are positive for both SMA and Aβ (**C**) and are considered autofluorescence artifacts rather than true SMA or Aβ deposition (*arrowheads*). Aβ, amyloid β; SMA, smooth muscle actin; NFT, neurofibrillary tangle; SP, senile plaque.

	Patient 1	Patient 2	Patient 3	Patient 4	Patient 5	Patient 6
Age at time of death, years (y)/ sex	74 y/ M	95 y/ M	67 y/ M	79 y/ F	78 y/ F	84 y/ M
APOE genotype[†]	3*3	3*3	3*3	3*3	3*3	4*4
Cardiovascular risk factor	HT	HT, Af, CHF	-	HT	HT, DLP	DM
**Clinical symptoms**						
Dementia	+ (bvFTD)	+	+	+	+	+
Intracranial Hemorrhage	-	-	-	-	+	-
**Brain MRI findings**						
Microbleeds	N.A.[‡,‡‡]	-	N.A.[§]	+	+	+
WM hyperintensity	N.A.[‡,‡‡]	+	+	+	+	+
EPVS	N.A.[‡]	+	-	+	+	+
**Pathological diagnosis (other than CAA)**	-	AD	AD MSA (OPC=SN)	AD PD (limbic)	AD	AD CPM, EPM, PD (limbic)
CAA type[9)]	2	2	2	1	1	1
CAA Thal stage[19)]	2	2	2	3	3	2
**CAA scoring[60)]**						
Parenchymal (F/O)	1/2	1/2	3/3	2/3	3/3	2/3
Meningeal (F/O)	2/3	2/2	2/3	2/3	3/3	2/3
Capillary (F/O)	0/0	0/0	0/0	1/1	1/1	1/1
Vasculopathy (F/O)	0/0	0/0	1/2	0/2	2/2	1/2
Aβ-positivity of CAA (F/O)*	low/ moderate	low/ moderate	moderate/ high	moderate/ high	high/ high	high/ high
Braak (NFT)/ Braak (SP)[57)]	III/C	IV/C	V/C	IV/C	V/C	VI/C
ADNC[58)]	A3B2C1	A2B2C3	A3B3C2	A3B3C3	A3B3C3	A3B3C3
Atherosclerosis/ Arteriolosclerosis[59)]	3/3	2/2	1/1	3/1	3/2	1/2

Table S1. Clinicopathological characteristics of patients with cerebral amyloid angiopathy.

+ and -, presence and absence, respectively; N.A., not available; HT, hypertension; Af, atrial fibrillation; CHF, congestive heart failure; DLP, dyslipidemia; DM, diabetes mellitus; bvFTD, behavioral variant frontotemporal dementia. CAA, cerebral amyloid angiopathy; EPVS: enlarged perivascular space in centrum semiovale. AD, Alzheimer's disease; MSA, multiple system atrophy; PD, Parkinson's disease; CPM, central pontine myelinolysis; EPM, extrapontine myelinolysis. F, frontal lobe; O, occipital lobe. Braak (NFT)/Braak (SP): Braak neurofibrillary tangle stage (I-VI)/neuritic plaque score (A-C; C = frequent). ADNC: AD neuropathologic change, A = Aβ deposition (Thal phase), B = NFT stage (Braak), C = CERAD neuritic plaque score (C = frequent). Atherosclerosis in the arterial circle of Willis, 3: severe, 2: moderate, 1: mild; arteriolosclerosis in the centrum semiovale, 3: severe, 2: moderate, 1: mild. *Aβ-positivity within blood vascular units, percentage of Aβ-positive vascular units to total vascular units per sample measured from the three-dimensional images acquired in this study (mild: <40 %; moderate: 40-60%; severe: >60%).†: APOE genotyping was performed using genomic DNA extracted from fresh-frozen occipital cortices, as reported previously (*61*). Five patients had APOE ε3/ε3 and one patient (Patient 6) had APOE ε4/ε4. ‡: Brain MRI data were not available. ‡‡: No low-density lesion in the white matter on brain CT scan. §: T2*-weighted MRI data were not available.

Movie S1. Distribution of Aβ in the vascular network in a sample from the low vascular Aβ load group revealed using Aβ and SMA immunostaining. This video shows a sample of the frontal lobe of Patient 2. Three-dimensional reconstruction of the vascular network. SMA (*yellow*) and Aβ (*blue*). The immunoreactivity of vascular smooth muscle cells is preserved in the cerebral arteries. The cortex contains numerous senile plaques. The numerous yellow spots observed in the videos represent neuronal lipofuscin characterized by strong autofluorescence.

Movie S2. Distribution of Aβ in the vascular network in a sample from the high vascular Aβ load group revealed using Aβ and SMA immunostaining. This video presents a sample of the frontal lobe of Patient 6. SMA (*yellow*) and Aβ (*blue*). Significant loss of vascular smooth muscle cells is evident in most vessels.

Movie S3. Analysis of the Aβ deposition pattern within vascular units. Replacement of vascular units in the brain parenchyma with "filaments".

This video presents a sample of the frontal lobe of Patient 6. SMA (*yellow*) and Aβ (*blue*). A schematic three-dimensional reconstruction of the Aβ-positive vascular network is provided, with Aβ-positive segments traced in *pink* and SMA-positive segments traced in *yellow*.

Movie S4. Vascular segments with SMA loss and Aβ deposition. This image presents a sample of the frontal lobe of Patient 6. SMA (*yellow*) and Aβ (*blue*). Vascular smooth muscle cells are lost in the vascular units of the brain surface and have been replaced by Aβ.

Movie S5. Analysis of perivascular plaque density. Replacement of perivascular senile plaques in the brain parenchyma with "spots". This image shows a sample of the frontal lobe of Patient 3. Traced filaments represent Aβ-positive (*red lines*) and Aβ-negative vascular units (*white lines*) with perivascular spots. Plaques around Aβ-positive vascular units (*orange spheres*) and Aβ-negative vascular units (*blue spheres*).

Movie S6. Serial tracing to verify the arterial identity of SMA-negative vascular segments. This movie shows cross-sectional views of a 3D-reconstructed Aβ-positive/SMA-negative vascular unit from a sample of the occipital lobe of Patient 1. SMA (*yellow*) and Aβ (*blue*). Some vessel segments appeared SMA-negative due to loss of SMA immunoreactivity; these were serially traced to adjacent upstream or downstream portions where SMA reappeared, confirming their anatomical continuity.

Movie S7. Identification of Aβ-positive/SMA-positive segments using 3D-to-2D cross-sectional views. This movie shows cross-sectional views of a 3D-reconstructed Aβ-positive/SMA negative vascular unit from a sample of the frontal lobe of Patient 4. SMA (*yellow*) and Aβ (*blue*). In Aβ-positive segments, the Aβ channel is temporarily turned off to evaluate the corresponding SMA signal. If SMA labeling is preserved and continuous along the same vascular trajectory in adjacent slices, the segment is classified as Aβ-positive/SMA-positive.

Data file S1. Vessel diameters for all of the vascular units analyzed (Excel file). This Excel file shows the actual measured external diameters of all the vascular units (Depth 0 segments) analyzed in this study. Aβ, amyloid β; $Aβ^+$, Aβ-positive; $Aβ^−$, Aβ-negative.

REFERENCES

1. T. Revesz, J. Ghiso, T. Lashley, G. Plant, A. Rostagno, B. Frangione, J. L. Holton, Cerebral amyloid angiopathies: A pathologic, biochemical, and genetic view. *J. Neuropathol. Exp. Neurol.* **62**, 885–898 (2003).

2. J. Attems, Sporadic cerebral amyloid angiopathy: Pathology, clinical implications, and possible pathomechanisms. *Acta Neuropathol.* **110**, 345–359 (2005).

3. A. Keable, K. Fenna, H. M. Yuen, D. A. Johnston, N. R. Smyth, C. Smith, R. Al-Shahi Salman, N. Samarasekera, J. A. R. Nicoll, J. Attems, R. N. Kalaria, R. O. Weller, R. O. Carare, Deposition of amyloid β in the walls of human leptomeningeal arteries in relation to perivascular drainage pathways in cerebral amyloid angiopathy. *Biochim. Biophys. Acta* **1862**, 1037–1046 (2016).

4. L. Szalardy, B. Fakan, R. Maszlag-Torok, E. Ferencz, Z. Reisz, B. L. Radics, S. Csizmadia, L. Szpisjak, A. Annus, D. Zadori, G. G. Kovacs, P. Klivenyi, Identifying diagnostic and prognostic factors in cerebral amyloid angiopathy-related inflammation: A systematic analysis of published and seven new cases. *Neuropathol. Appl. Neurobiol.* **50**, e12946 (2024).

5. A. Viswanathan, S. M. Greenberg, Cerebral amyloid angiopathy in the elderly. *Ann. Neurol.* **70**, 871–880 (2011).

6. S. Budd Haeberlein, P. S. Aisen, F. Barkhof, S. Chalkias, T. Chen, S. Cohen, G. Dent, O. Hansson, K. Harrison, C. von Hehn, T. Iwatsubo, C. Mallinckrodt, C. J. Mummery, K. K. Muralidharan, I. Nestorov, L. Nisenbaum, R. Rajagovindan, L. Skordos, Y. Tian, C. H. van Dyck, B. Vellas, S. Wu, Y. Zhu, A. Sandrock, Two randomized phase 3 studies of aducanumab in early Alzheimer’s disease. *J. Prev Alzheimers Dis.* **9**, 197–210 (2022).

7. N. J. Reish, P. Jamshidi, B. Stamm, M. E. Flanagan, E. Sugg, M. Tang, K. L. Donohue, M. McCord, C. Krumpelman, M.-M. Mesulam, R. Castellani, S. H.-Y. Chou, Multiple cerebral hemorrhages in a patient receiving lecanemab and treated with t-PA for stroke. *N. Engl. J. Med.* **388**, 478–479 (2023).

8. E. Solopova, W. Romero-Fernandez, H. Harmsen, L. Ventura-Antunes, E. Wang, A. Shostak, J. Maldonado, M. J. Donahue, D. Schultz, T. M. Coyne, A. Charidimou, M. Schrag, Fatal iatrogenic cerebral β-amyloid-related arteritis in a woman treated with lecanemab for Alzheimer's disease. *Nat. Commun.* **14**, 8220 (2023).

9. D. R. Thal, E. Ghebremedhin, U. Rüb, H. Yamaguchi, K. D. Tredici, H. Braak, Two types of sporadic cerebral amyloid angiopathy. *J. Neuropathol. Exp. Neurol.* **61**, 282–293 (2002).

10. C. Carlson, E. Siemers, A. Hake, M. Case, R. Hayduk, J. Suhy, J. Oh, J. Barakos, Amyloid-related imaging abnormalities from trials of solanezumab for Alzheimer's disease. *Alzheimers Dement.* **2**, 75–85 (2016).

11. H. V. Vinters, Cerebral amyloid angiopathy: A critical review. *Stroke* **18**, 311–324 (1987).

12. M. Kawai, R. N. Kalaria, P. Cras, S. L. Siedlak, M. E. Velasco, E. R. Shelton, H. W. Chan, B. D. Greenberg, G. Perry, Degeneration of vascular muscle cells in cerebral amyloid angiopathy of Alzheimer disease. *Brain Res.* **623**, 142–146 (1993).

13. J. P. Vonsattel, R. H. Myers, E. T. Hedley-Whyte, A. H. Ropper, E. D. Bird, E. P. Richardson Jr., Cerebral amyloid angiopathy without and with cerebral hemorrhages: A comparative histological study. *Ann. Neurol.* **30**, 637–649 (1991).

14. A. Charidimou, G. Boulouis, M. E. Gurol, C. Ayata, B. J. Bacskai, M. P. Frosch, A. Viswanathan, S. M. Greenberg, Emerging concepts in sporadic cerebral amyloid angiopathy. *Brain* **140**, 1829–1850 (2017).

15. E. A. Koemans, J. P. Chhatwal, S. J. van Veluw, E. S. van Etten, M. J. P. van Osch, M. A. A. van Walderveen, H. R. Sohrabi, M. G. Kozberg, Z. Shirzadi, G. M. Terwindt, M. A. van Buchem, E. E. Smith, D. J. Werring, R. N. Martins, M. J. H. Wermer, S. M. Greenberg, Progression of cerebral amyloid angiopathy: A pathophysiological framework. *Lancet Neurol.* **22**, 632–642 (2023).

16. E. Richard, A. Carrano, J. J. Hoozemans, J. van Horssen, E. S. van Haastert, L. S. Eurelings, H. E. de Vries, D. R. Thal, P. Eikelenboom, W. A. van Gool, A. J. M. Rozemuller, Characteristics of

dyshoric capillary cerebral amyloid angiopathy. *J. Neuropathol. Exp. Neurol.* **69**, 1158–1167 (2010).

17. D. M. Kamara, U. Gangishetti, M. Gearing, M. Willis-Parker, L. Zhao, W. T. Hu, L. C. Walker. Cerebral amyloid angiopathy: Similarity in African-Americans and Caucasians with Alzheimer's disease. *J. Alzheimer's Dis* **62**, 1815–1826 (2018).

18. L. Szalárdy, S. Lee, A. Kim, G. G. Kovacs. Distinct cerebral amyloid angiopathy patterns in adult Down syndrome. *J. Neurol. Sci.***476**, 123601 (2025).

19. D. R. Thal, E. Ghebremedhin, M. Orantes, O. D. Wiestler, Vascular pathology in Alzheimer disease: Correlation of cerebral amyloid angiopathy and arteriosclerosis/lipohyalinosis with cognitive decline. *J. Neuropathol. Exp. Neurol.* **62**, 1287–1301 (2003).

20. S. Takeda, K. Yamazaki, T. Miyakawa, K. Onda, Cerebral amyloid angiopathy initially occurs in the meningeal vessels. *Neuropathology* **37**, 502–508 (2017).

21. E. T. Zhang, H. K. Richards, S. Kida, R. O. Weller, Directional and compartmentalised drainage of interstitial fluid and cerebrospinal fluid from the rat brain. *Acta Neuropathol.* **83**, 233–239 (1992).

22. S. Kida, A. Pantazis, R. O. Weller, CSF drains directly from the subarachnoid space into nasal lymphatics in the rat: Anatomy, histology and immunological significance. *Neuropathol. Appl. Neurobiol.* **19**, 480–488 (1993).

23. M. E. Calhoun, P. Burgermeister, A L Phinney, M. Stalder, M. Tolnay, K. H. Wiederhold, D. Abramowski, C Sturchler-Pierrat, B. Sommer, M. Staufenbiel, M. Jucker, Neuronal overexpression of mutant amyloid precursor protein results in prominent deposition of cerebrovascular amyloid. *Proc. Natl. Acad. Sci. U.S.A.* **96**, 14088–14093 (1999).

24. R. O. Weller, E. Djuanda, H.-Y. Yow, R. O. Carare, Lymphatic drainage of the brain and the pathophysiology of neurological disease. *Acta Neuropathol.* **117**, 1–14 (2008).

25. S. M. Greenberg, B. J. Bacskai, M. Hernandez-Guillamon, J. Pruzin, R. Sperling, S. J. van Veluw, Cerebral amyloid angiopathy and Alzheimer disease—One peptide, two pathways. *Nat. Rev. Neurol.* **16**, 30–42 (2020).

26. J. M. Tarasoff-Conway, R. O. Carare, R. S. Osorio, L. Glodzik, T. Butler, E. Fieremans, L. Axel, H. Rusinek, C. Nicholson, B. V. Zlokovic, B. Frangione, K. Blennow, J. Ménard, H. Zetterberg, T. Wisniewski, M. J. de Leon, Clearance systems in the brain-implications for Alzheimer disease. *Nat. Rev. Neurol.* **11**, 457–470 (2015).

27. A. Rovelet-Lecrux, D. Hannequin, G. Raux, N. Le Meur, A. Laquerrière, A. Vital, C. Dumanchin, S. Feuillette, A. Brice, M. Vercelletto, F. Dubas, T. Frebourg, D. Campion, APP locus duplication causes autosomal dominant early-onset Alzheimer disease with cerebral amyloid angiopathy. *Nat. Genet.* **38**, 24–26 (2006).

28. E. Head, M. J. Phelan, E. Doran, R. C. Kim, W. W. Poon, F. A. Schmitt, I. T. Lott, Cerebrovascular pathology in Down syndrome and Alzheimer disease. *Acta Neuropathol. Commun.* **5**, 93 (2017).

29. D. A. Loeffler, Approaches for increasing cerebral efflux of amyloid-β in experimental systems. *J. Alzheimer's Dis* **100**, 379–411 (2024).

30. R. O. Carare, M. Bernardes-Silva, T. A. Newman, A. M. Page, J. A. R. Nicoll, V. H. Perry, R. O. Weller, Solutes, but not cells, drain from the brain parenchyma along basement membranes of capillaries and arteries: Significance for cerebral amyloid angiopathy and neuroimmunology. *Neuropathol. Appl. Neurobiol.* **34**, 131–144 (2008).

31. C. A. Hawkes, W. Härtig, J. Kacza, R. Schliebs, R. O. Weller, J. A. R. Nicoll, R. O. Carare, Perivascular drainage of solutes is impaired in the ageing mouse brain and in the presence of cerebral amyloid angiopathy. *Acta Neuropathol.* **121**, 431–443 (2011).

32. A. W. Morris, M. M. Sharp, N. J. Albargothy, R. Fernandes, C. A. Hawkes, A. Verma, R. O. Weller, R. O. Carare, Vascular basement membranes as pathways for the passage of fluid into and out of the brain. *Acta Neuropathol.* **131**, 725–736 (2016).

33. N. J. Albargothy, D. A. Johnston, M. J. MacGregor-Sharp, R. O. Weller, A. Verma, C. A. Hawkes, R. O. Carare, Convective influx/glymphatic system: Tracers injected into the CSF enter and leave the brain along separate periarterial basement membrane pathways. *Acta Neuropathol.* **136**, 139–152 (2018).

34. A. D. Joshi, M. J. Pontecorvo, M. Lu, D. M. Skovronsky, M. A. Mintun, M. D. Devous Sr., A semiautomated method for quantification of F18 florbetapir PET images. *J. Nucl. Med.* **56**, 1736–1741 (2015).

35. W. E. Klunk, R. A. Koeppe, J. C. Price, T. L. Benzinger, M. D. Devous Sr., W. J. Jagust, K. A. Johnson, C. A. Mathis, D. Minhas, M. J. Pontecorvo, C. C. Rowe, D. M. Skovronsky, M. A. Mintun, The Centiloid Project: Standardizing quantitative amyloid plaque estimation by PET. *Alzheimers Dement.* **11**, 1–15.e4 (2015).

36. K. Tainaka, T. C. Murakami, E. A. Susaki, C. Shimizu, R. Saito, K. Takahashi, A. Hayashi-Takagi, H. Sekiya, Y. Arima, S. Nojima, M. Ikemura, T. Ushiku, Y. Shimizu, M. Murakami, K. F. Tanaka, M. Iino, H. Kasai, T. Sasaoka, K. Kobayashi, K. Miyazono, E. Morii, T. Isa, M. Fukayama, A. Kakita, H. R. Ueda, Chemical landscape for tissue clearing based on hydrophilic reagents. *Cell Rep.* **24**, 2196–2210.e9 (2018).

37. M. Inoue, R. Saito, A. Kakita, K. Tainaka, Rapid chemical clearing of white matter in the post-mortem human brain by 1,2-hexanediol delipidation. *Bioorg. Med. Chem. Lett.* **29**, 1886–1890 (2019).

38. A. Gilvesy, E. Husen, Z. Magloczky, O. Mihaly, T. Hortobágyi, S. Kanatani, H. Heinsen, N. Renier, T. Hökfelt, J. Mulder, M. Uhlen, G. G. Kovacs, C. Adori, Spatiotemporal characterization of cellular tau pathology in the human locus coeruleus-pericoerulear complex by three-dimensional imaging. *Acta Neuropathol.* **144**, 651–676 (2022).

39. T. Liebmann, N. Renier, K. Bettayeb, P. Greengard, M. Tessier-Lavigne, M. Flajolet, Three-dimensional study of Alzheimer’s disease hallmarks using the iDISCO clearing method. *Cell Rep.* **16**, 1138–1152 (2016).

40. S. B. Domnitz, E. M. Robbins, A. W. Hoang, M. Garcia-Alloza, B. T. Hyman, G William Rebeck, S. M. Greenberg, B. J. Bacskai, M. P. Frosch, Progression of cerebral amyloid angiopathy in transgenic mouse models of Alzheimer disease. *J. Neuropathol. Exp. Neurol.* **64**, 588–594 (2005).

41. E. M. Robbins, R. A. Betensky, S. B. Domnitz, S. M. Purcell, M. Garcia-Alloza, C. Greenberg, G. W. Rebeck, B. T. Hyman, S. M. Greenberg, M. P. Frosch, B. J. Bacskai, Kinetics of cerebral amyloid angiopathy progression in a transgenic mouse model of Alzheimer disease. *J. Neurosci.* **26**, 365–371 (2006).

42. H. M. Wisniewski, J. Wegiel, β-amyloid formation by myocytes of leptomeningeal vessels. *Acta Neuropathol.* **87**, 233–241 (1994).

43. Y. Shinkai, M. Yoshimura, M. Morishima-Kawashima, Y. Ito, H. Shimada, K. Yanagisawa, Y. Ihara, Amyloid β-protein deposition in the leptomeninges and cerebral cortex. *Ann. Neurol.* **42**, 899–908 (1997).

44. M. K. Rasmussen, H. Mestre, M. Nedergaard, The glymphatic pathway in neurological disorders. *Lancet Neurol.* **17**, 1016–1024 (2018).

45. A. Maeda, M. Yamada, Y. Itoh, E. Otomo, M. Hayakawa, T. Miyatake, Computer-assisted three-dimensional image analysis of cerebral amyloid angiopathy. *Stroke* **24**, 1857–1864 (1993).

46. S. H. Kim, J. H. Ahn, H. Yang, P. Lee, G. Y. Koh, Cerebral amyloid angiopathy aggravates perivascular clearance impairment in an Alzheimer's disease mouse model. *Acta Neuropathol. Commun.* **8**, 181 (2020).

47. F. Reina-De La Torre, A. Rodriguez-Baeza, J. Sahuquillo-Barris, Morphological characteristics and distribution pattern of the arterial vessels in human cerebral cortex: A scanning electron microscope study. *Anat. Rec.* **251**, 87–96 (1998).

48. A. Biffi, S. M. Greenberg, Cerebral amyloid angiopathy: A systematic review. *J. Clin. Neurol.* **7**, 1–9 (2011).

49. T. Mendel, T. Wierzba-Bobrowicz, T. Stępień, G. M. Szpak, β-amyloid deposits in veins in patients with cerebral amyloid angiopathy and intracerebral haemorrhage. *Folia Neuropathol.* **51**, 120–126 (2013).

50. M. Yamada, H. Tsukagoshi, E. Otomo, M. Hayakawa, Cerebral amyloid angiopathy in the aged. *J. Neurol.* **234**, 371–376 (1987).

51. R. J. Ellis, J. M. Olichney, L. J. Thal, S. S. Mirra, J. C. Morris, D. Beekly, A. Heyman, Cerebral amyloid angiopathy in the brains of patients with Alzheimer's disease: The CERAD experience, Part XV. *Neurology* **46**, 1592–1596 (1996).

52. J. Tian, J. Shi, K. Bailey, D. M. A. Mann, Negative association between amyloid plaques and cerebral amyloid angiopathy in Alzheimer's disease. *Neurosci. Lett.* **352**, 137–140 (2003).

53. N. Allen, A. C. Robinson, J. Snowden, Y. S. Davidson, D. M. A. Mann, Patterns of cerebral amyloid angiopathy define histopathological phenotypes in Alzheimer's disease. *Neuropathol. Appl. Neurobiol.* **40**, 136–148 (2014).

54. J. S. Rabin, E. Nichols, R. L. Joie, K. B. Casaletto, P. Palta, K. Dams-O'Connor, R. G. Kumar, K. M. George, C. L. Satizabal, J. A. Schneider, J. Pa, A. M. Brickman, Cerebral amyloid angiopathy interacts with neuritic amyloid plaques to promote tau and cognitive decline. *Brain* **145**, 2823–2833 (2022).

55. T. J. Esparza, N. C. Wildburger, H. Jiang, M. Gangolli, N. J. Cairns, R. J. Bateman, D. L. Brody, Soluble amyloid-beta aggregates from human Alzheimer's disease brains. *Sci. Rep.* **6**, 38187 (2016).

56. T. J. Montine, C. H. Phelps, T. G. Beach, E. H. Bigio, S. D. Cairns, S. H. Dickson, K. H. Duyckaerts, C. Frosch, P. T. Masliah, R. C. Mirra, W. S. Nelson, J. A. Schneider, K. A. Thal, J. Q. Trojanowski, H. V. Vinters, B. T. Hyman, National Institute on Aging, Alzheimer's Association, National institute on Aging-Alzheimer's association guidelines for the neuropathologic assessment of Alzheimer's disease: A practical approach. *Acta Neuropathol.* **123**, 1–11 (2012).

57. H. Braak, E. Braak, Neuropathological stageing of Alzheimer-related changes, *Acta Neuropathol.* **82**, 239–259 (1991).

58. I. G. McKeith, B. F. Boeve, D. W. Dickson, G. Halliday, J.-P. Taylor, D. Weintraub, D. Aarsland, J. Galvin, J. Attems, C. G. Ballard, A. Bayston, T. G. Beach, F. Blanc, N. Bohnen, L. Bonanni, J. Bras, P. Brundin, D. Burn, A. Chen-Plotkin, J. E. Duda, O. El-Agnaf, H. Feldman, T. J. Ferman, D. Ffytche, H. Fujishiro, D. Galasko, J. G. Goldman, S. N. Gomperts, N. R. Graff-Radford, L. S. Honig, A. Iranzo, K. Kantarci, D. Kaufer, W. Kukull, V. M. Y. Lee, J. B. Leverenz, S. Lewis, C. Lippa, A. Lunde, M. Masellis, E. Masliah, P. M. Lean, B. Mollenhauer, T. J. Montine, E. Moreno, E. Mori, M. Murray, J. T. O'Brien, S. Orimo, R. B. Postuma, S. Ramaswamy, O. A. Ross, D. P. Salmon, A. Singleton, A. Taylor, A. Thomas, P. Tiraboschi, J. B. Toledo, J. Q. Trojanowski, D. Tsuang, Z. Walker, M. Yamada, K. Kosaka, Diagnosis and management of dementia with Lewy bodies: Fourth consensus report of the DLB Consortium. *Neurology* **89**, 88–100 (2017).

59. V. Deramecourt, J. Y. Slade, A. E. Oakley, R. H. Perry, P. G. Ince, C.-A. Maurage, R. N. Kalaria, Staging and natural history of cerebrovascular pathology in dementia. *Neurology* **78**, 1043–1050 (2012).

60. S. Love, K. Chalmers, P. Ince, M. Esiri, J. Attems, K. Jellinger, M. Yamada, M. M. Carron, T. Minett, F. Matthews, S. Greenberg, D. Mann, P. G. Kehoe, Development, appraisal, validation and implementation of a consensus protocol for the assessment of cerebral amyloid angiopathy in post-mortem brain tissue. *Am. J. Neurodegener. Dis.* **3**, 19–32 (2014).

61. T. Ikeuchi, M. Kanda, H. Kitamura, F. Morikawa, S. Toru, C. Nishimura, K. Kasuga, T. Tokutake, T. Takahashi, Y. Kuroha, N. Miyazawa, S. Tanaka, K. Utsumi, K. Ono, S. Yano, T. Hamano, S. Naruse, R. Yajima, N. Kawashima, C. Kaneko, H. Tachibana, Y. Yano, Y. Kato, S. Toue, H. Jinzu, A. Kitamura, Y. Yokoyama, E. Kaneko, M. Yamakado, K. Nagao, Decreased circulating branched-chain amino acids are associated with development of Alzheimer's disease in elderly individuals with mild cognitive impairment. *Front. Nutr.* **9**, 1040476 (2022).
